# Combined metagenomic and phenomic approaches identify a novel salt tolerance gene from the human gut microbiome

**DOI:** 10.3389/fmicb.2014.00189

**Published:** 2014-04-29

**Authors:** Eamonn P. Culligan, Julian R. Marchesi, Colin Hill, Roy D. Sleator

**Affiliations:** ^1^Alimentary Pharmabiotic Centre, Biosciences Institute, University College CorkCork, Ireland; ^2^School of Microbiology, University College CorkCork, Ireland; ^3^Cardiff School of Biosciences, Cardiff UniversityCardiff, UK; ^4^Department of Surgery and Cancer, Centre for Digestive and Gut Health, Imperial College LondonLondon, UK; ^5^Department of Biological Sciences, Cork Institute of TechnologyCork, Ireland

**Keywords:** metagenomics, functional metagenomics, gut microbiome, microbiota, salt tolerance, BIOLOG, phenotype microarray, transcriptional regulator

## Abstract

In the current study, a number of salt-tolerant clones previously isolated from a human gut metagenomic library were screened using Phenotype MicroArray (PM) technology to assess their functional capacity. PM's can be used to study gene function, pathogenicity, metabolic capacity and identify drug targets using a series of specialized microtitre plate assays, where each well of the microtitre plate contains a different set of conditions and tests a different phenotype. Cellular respiration is monitored colorimetrically by the reduction of a tetrazolium dye. One clone, SMG 9, was found to be positive for utilization/transport of L-carnitine (a well-characterized osmoprotectant) in the presence of 6% w/v sodium chloride (NaCl). Subsequent experiments revealed a significant growth advantage in minimal media containing NaCl and L-carnitine. Fosmid sequencing revealed putative candidate genes responsible for the phenotype. Subsequent cloning of two genes did not replicate the L-carnitine-associated phenotype, although one of the genes, a σ^54^-dependent transcriptional regulator, did confer salt tolerance to *Escherichia coli* when expressed in isolation. The original clone, SMG 9, was subsequently found to have lost the original observed phenotype upon further investigation. Nevertheless, this study demonstrates the usefulness of a phenomic approach to assign a functional role to metagenome-derived clones.

## Introduction

The ability to adapt to and tolerate increases in extracellular osmolarity is an important characteristic that enables bacteria to survive in stressful environments. Increased osmolarity, caused by sodium chloride (NaCl) for example, initiates a phased response in bacteria. Firstly during the primary response, potassium ions are rapidly accumulated within the bacterial cell to offset the detrimental effects of water loss and influx of toxic sodium and chloride ions (Sleator and Hill, 2002; Epstein, 2003). Once the cell has been stabilized, the secondary response begins and involves the synthesis or, more often the more energetically favorable, uptake of osmoprotectant compounds (also termed compatible solutes or osmolytes). Osmoprotectants are compatible with cellular functions and can accumulate to very high concentrations within the cell and function to protect proteins and to restore cell volume and thus, turgor pressure (Kempf and Bremer, 1998; Sleator and Hill, 2002; Kunte, 2006). Osmoprotectants can be grouped broadly as amino acids, polyols, sugars, trimethyl- and quaternary-ammonium compounds and their derivatives (Kempf and Bremer, 1998). The most widely utilized and best characterized osmoprotectants are glycine betaine, carnitine, proline, and ectoine. Numerous studies have shown carnitine to be important not only for salt tolerance, but also for survival *in vivo* and pathogenesis of infection (Sleator et al., 2001; Wemekamp-Kamphuis et al., 2004). Carnitine is also found abundantly in animal tissues and red meat and is an important compound in the host environment; for the human pathogen *Listeria monocytogenes*, carnitine and its uptake system OpuC are critical for infection in mice (Sleator et al., 2003). In addition to its osmoprotective properties, carnitine may also be catabolised as a carbon or nitrogen source to generate energy (Wargo and Hogan, 2009).

The emergence of “omics” technologies, an umbrella term to include analyses such as genomics, metagenomics, transcriptomics, proteomics, metabolomics, and phenomics to name a few, have been used to gain valuable information about the functions and interactions of various biological systems as a whole and can provide more information than more traditional and reductive approaches to biological problems. Sequence-based and functional metagenomic approaches have led to the discovery of many novel and diverse genes (Beja et al., 2000; Gillespie et al., 2002; Banik and Brady, 2008; Culligan et al., 2013; Yoon et al., 2013). While identifying clones which display a specific phenotype through functional metagenomic screening yields worthwhile results, characterizing the functional mechanisms responsible for the observed phenotype can be sometimes difficult owing to the fact that a large proportion of metagenome derived genes will be annotated as hypothetical proteins or have no known function or homology to existing proteins (Bork, 2000; Qin et al., 2010). Phenomic approaches can be used to study hundreds of phenotypic profiles of different bacterial strains concurrently. Comparing phenomic profiles of wild-type and mutant derivatives or host strains and clones identified through metagenomic screening can reveal differences between strains relating to gene function, pathogenicity and metabolism for example (Bochner et al., 2001; Bochner, 2009).

**Figure 1:**
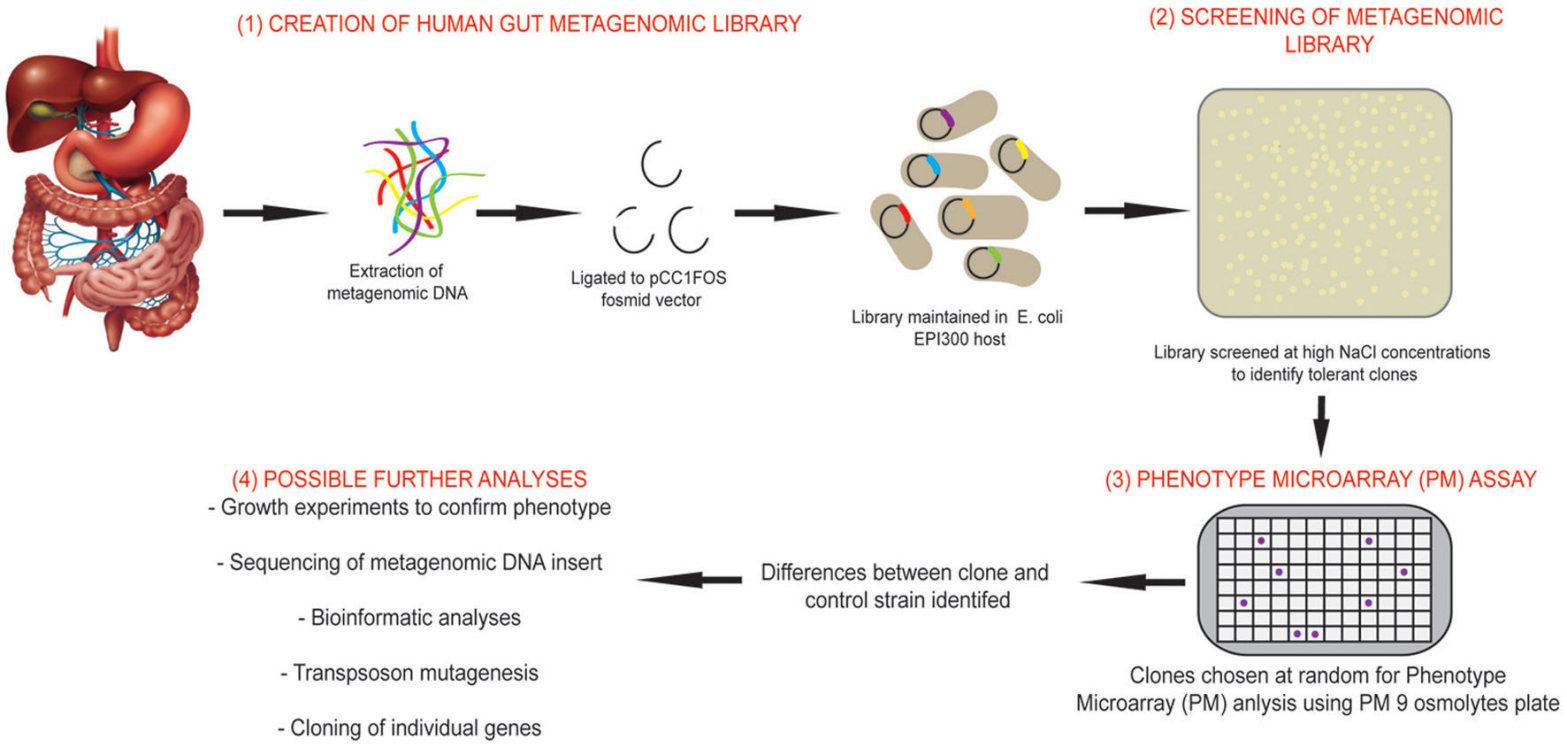
**Overview of the study design using combined functional metagenomic and phenomic approaches**. An overview of the study design and experimental process incorporating metagenomic library creation and screening, identification of salt tolerant clones, comparison of clone to control strain using Phenotypic Microarray (PM) assay and further analyses that may be performed to identify novel genes.

With this in mind, we have utilized combined metagenomic and phenomic approaches in this study to characterize a salt tolerant clone identified from a human gut microbiome metagenomic fosmid library. An overview of the study design using this combined approach can be seen in Figure 1.The BIOLOG phenotype microarray (PM) system was used to compare phenotypes between metagenomic clones and cloning host (carrying empty fosmid vector). PM plates measure cellular respiration colorimetrically via reduction of a tetrazolium dye with electrons from NADH generated during the process of respiration. Strongly metabolized substrates generate a more intense purple color which is recorded with a camera on the Omnilog instrument. If desired, thousands of phenotypes may be monitored simultaneously using the different available PM plates which can be grouped as those that measure carbon, nitrogen, phosphorous and sulfur metabolism, response to different pH conditions, osmolytes and chemicals such as anitbiotics. The full range of PM plates and their constituents, for investigation of bacterial phenotypes can be found at: http://www.biolog.com/products-static/phenotype_microbial_cells_use.php

As our primary interest is salt tolerance we used the PM osmolyte plate (PM9) for analysis. The PM screen indicated that clone SMG 9 was positive for L-carnitine utilization in the presence of 6% w/v NaCl. Sequencing of the fosmid insert and cloning of two genes identified a novel salt tolerance gene, but did not replicate the carnitine-associated phenotype originally observed. Subsequent investigation revealed SMG 9 had lost this phenotype. Notwithstanding this phenomenon, this study demonstrates the usefulness of a phenomic approach to assign a functional role to metagenomic library-derived clones.

## Materials and methods

### Bacterial strains and growth conditions

Bacterial strains, plasmids, and oligonucleotide primers (Eurofins, MWG Operon, Germany) used in this study are listed in Table 1. *Escherichia coli* EPI300::pCC1FOS (Epicentre Biotechnologies, Madison, WI, USA) was grown in Luria-Bertani (LB) medium containing 12.5 μg/ml chloramphenicol (Cm). *E. coli* MKH13 was grown in LB medium and in LB medium supplemented with 20 μg/ml Cm for strains transformed with the plasmid pCI372. LB media was supplemented with 1.5% w/v agar when required. All overnight cultures were grown at 37°C with shaking.

**Table 1:** **Bacterial strains, plasmids and oligonucleotide primers used in this study**.

**Strain or plasmid**	**Genotype or characteristic(s)**	**Source or references**
**STRAINS**		
*E. coli* EPI300	F^−^*mcrA* Δ(*mrr-hsd*RMS*-mcrBC*) Φ 80d*lacZ*ΔM15 Δ*lac*X74 *recA*1 *endA*1 *araD*139 Δ(*ara, leu*)7697 *galU galK* λ ^−^ *rpsL nupG trfA dhfr*; high-transformation efficiency of large DNA	Epicentre Biotechnologies, Madison, WI, USA
*E.coli* MKH13	MC4100*Δ*(*putPA*)101D(*proP*)2D(*proU*)	Haardt et al., 1995
*E. coli* MKH13::pCI372-*mfsT*	MKH13 containing pCI372 with *mfsT* gene from SMG 9 (similar to *Bacteroides* sp. CAG:545)	This study
*E. coli* MKH13::pCI372-*sdtR*	MKH13 containing pCI372 with *sdtR* gene from SMG 9 (similar to *Bacteroides* sp. CAG:545)	This study
*E. coli* EPI300::pCI372-*mfsT*	EPI300 containing pCI372 with *mfsT* gene from SMG 9 (similar to *Bacteroides* sp. CAG:545)	This study
*E. coli* EPI300::pCI372-*sdtR*	EPI300 containing pCI372 with *sdtR* gene from SMG 9 (similar to *Bacteroides* sp. CAG:545)	This study
**PLASMIDS**		
pCI372	Shuttle vector between *E. coli* and *L. lactis*, Cm^*R*^	Hayes et al., 1990
pCC1FOS	Fosmid cloning vector, Cm^*R*^	Epicentre Biotechnologies, Madison, WI, USA
**PRIMERS**		
pCI372 FP	CGGGAAGCTAGAGTAAGTAG	This study
pCI372 RP	CCTCTCGGTTATGAGTTAG	This study
*mfsT* FP	AAAACTGCAGCGGACTCGTGGTGGATGAC	This study
*mfsT* RP	GCTCTAGACAAACACCTTGGTGTCATTAGC	This study
*sdtR* FP	AAAAGTCGACCGGCTGTATGGAAGTTCCTG	This study
*sdtR* RP	GCTCTAGAGCCTCCAAATTTTGCACCAAG	This study

*FP, forward primer; RP, reverse primer; CmR, chlorpamphenicol resistance; Restriction enzyme cut sites are underlined; PstI, CTGCAG; XbaI, TCTAGA; SalI, GTCGAC*.

### Metagenomic library construction and screening

A previously constructed fosmid clone library (Jones and Marchesi, 2007; Jones et al., 2008), created from metagenomic DNA isolated from a fecal sample from a healthy 26 year old Caucasian male was used to screen for salt-tolerant clones. The library was screened as outlined previously (Culligan et al., 2012). Briefly, a total of 23,040 clones from the library were screened on LB agar supplemented with 6.5% (w/v) NaCl (a concentration which inhibits the growth of the cloning host, *E. coli* EPI300) and 12.5 μg/ml Cm using a Genetix QPix 2 XT colony picking/gridding robotics platform to identify clones with an increased salt tolerance phenotype compared to the cloning host (*E. coli* EPI300) carrying an empty fosmid vector (pCC1FOS). Identification of any salt tolerant clones will therefore most likely be due to a gene (or genes) present on the metagenomic insert from the human gut microbiota. Clones were gridded onto Q-trays (Genetix) using the robotics platform. Q-trays were incubated at 37°C for 3 days and checked twice daily for growth of likely salt-tolerant clones. Salt-tolerant clones were subsequently replica plated onto LB agar containing 12.5 μg/ml Cm and 6.5% NaCl and onto LB containing 12.5 μg/ml Cm, but without 6.5% NaCl (which represented a positive control plate). Each salt tolerant clone identified was streaked on LB agar + 12.5 μg/ml Cm to ensure a pure culture and all clones were maintained as glycerol stocks at −80°C.

### Phenotype microarray (PM) assay

The PM9 osmolytes microplate was used to compare the cellular phenotypes of SMG 9 and the cloning host, *E. coli* EPI300::pCC1FOS (containing an empty fosmid vector) under 96 different conditions. Preparation of the different IF (Inoculating Fluids; proprietary formulation supplied by BIOLOG) solutions and inoculation of the PM plates was performed according to the BIOLOG PM protocol for *E. coli* and other Gram negative bacteria. Briefly, IF-0 solution was prepared by adding 25 ml of sterile water to 125 ml of 1.2× IF-0. IF-0 + dye mix A solution was prepared by adding 1.8 ml of dye mix A and 23.2 ml of sterile water to another bottle containing 125 ml of 1.2× IF-0. IF-10 solution was prepared by adding 1.5 ml of dye mix A and 23.5 ml of sterile water to a bottle containing 125 ml of 1.2× IF-10. *E. coli* strains were grown overnight on LB agar at 37°C by streaking from a frozen stock. Cells were sub-cultured by streaking again on LB agar and grown overnight again. Isolated colonies were removed from the agar plate using a sterile swab and added to a tube containing 16 ml of IF-0 solution until a cell suspension of 42% T (transmittance) was achieved using the BIOLOG Turbidimeter. 15 ml of this 42% T solution was diluted in 75 ml of IF-0 + dye mix A to achieve 85% T. 600 μl of the 85% T cell suspension solution was added to 120 ml of IF-10 + dye mix A. Finally, 100 μl of the final cell suspension was inoculated to each well of the PM 9 microplate. Plates were incubated at 37°C for 24 h in the Omnilog plate reader (BIOLOG).

### Fosmid sequencing and analysis

Fosmid DNA was isolated from SMG 9 as described above to a concentration >200 ng/μl (approximately 5 μg in total). Sequencing of the full fosmid insert of SMG 9 was performed by GATC Biotech (Konstanz, Germany) using 454-pyrosequencing on a titanium mini-run of the Roche GS-FLX platform, achieving approximately 65-fold coverage. Sequencing reads were assembled into a single contig by GATC Biotech. The retrieved sequence was analyzed using the FGENESB software program (Softberry) to identify putative open reading frames and translated nucleotide sequences were subjected to BLASTP analysis to assign putative functions to the encoded proteins and identify homologous sequences from the National Centre for Biotechnology Information (NCBI; http://www.ncbi.nlm.nih.gov/blast/Blast.cgi). The full fosmid insert sequence of SMG 9 was submitted to GenBank and assigned the following accession number; KJ524644.

### DNA manipulations and cloning of *mfsT* and *sdtR* genes

Extraction of fosmids containing metagenomic DNA: 5 ml of bacterial culture was grown overnight with 12.5 μg/ml Cm. One millilitre of culture was used to inoculate 4 ml of fresh LB broth. To this, 5 μl of 1000× Copy Control Induction solution (Epicentre Biotechnologies) and 12.5 μg/ml Cm were added. The mixture was incubated at 37°C for 5 h with vigorous shaking (200–250 rpm) to ensure maximum aeration. Cells were harvested from the whole 5 ml of induced culture by centrifuging at 2100 × *g* for 12 min. Qiagen QIAprep Spin mini-prep kit was used to extract fosmids as per manufacturer's instructions. PCR products were purified with a Qiagen PCR purification kit and digested with *XbaI* and *PstI* (Roche Applied Science) for *mfsT* and with *SalI* and *XbaI* for *sdtR*, followed by ligation using the FastLink DNA ligase kit (Epicentre Biotechnologies) to similarly digested plasmid pCI372. Electrocompetent *E. coli* MKH13 and *E. coli* EPI300 wer*e* transformed with the ligation mixture and plated on LB agar plates containing 20 μg/ml Cm for selection. Colony PCR was performed on all resistant transformants using primers across the multiple cloning site (MCS) of pCI372 to confirm the presence and size of the insert.

### Confirmation tests for observed phenotype

Growth experiments were performed in defined M9 minimal media (M9MM) (Fluka) to confirm the observed phenotype. Single isolated colonies of SMG 9 and EPI300::pCC1FOS were grown overnight in M9MM (containing final concentrations of; D-glucose (0.4%), Bacto™ casamino acids (w/v 0.2%) (Becton, Dickinson and Co, Sparks, MD, USA), magnesium sulfate (MgSO_4_) (2 mM), calcium chloride (CaCl_2_) (0.1 mM) and 12.5 μg/ml Cm). Reagents were purchased from Sigma Aldrich (St. Louis, MO, USA) unless otherwise stated. Cells were harvested by centrifugation, washed in ¼ strength Ringers solution and resuspended in fresh M9MM. A 2% v/v inoculum was sub-cultured in fresh M9MM containing various concentrations of sodium chloride (0–8% w/v NaCl) and 1 mM of L-carnitine when required. Triplicate wells of a 96-well micro-titre plate were inoculated with 200 μl of the appropriate cell suspension. Plates were incubated at 37°C for 24–48 h in an automated spectrophotometer (Tecan Genios) which recorded the optical density at 595 nm (OD_595 nm_) every hour. After 48 h the data was retrieved and analyzed using the Magellan 3 software program and graphs were created with Sigma Plot 10.0 (Systat Software Inc, London, UK). Results are presented as the average of triplicate experiments, with error bars being representative of the standard error of the mean (SEM).

## Results

### Screening of metagenomic library

Approximately 23,000 clones from a metagenomic fosmid library from the human gut microbiome were screened previously and resulted in the identification of 53 salt tolerant clones which could grow on LB agar supplemented with 6.5% NaCl (a concentration which inhibits growth of the cloning host, *E. coli* EPI300::pCC1FOS) (Culligan et al., 2012). The salt tolerant clones identified were annotated as SMG 1-53 (Salt MetaGenome 1–53).

### PM osmolyte plate assay

**Figure 2:**
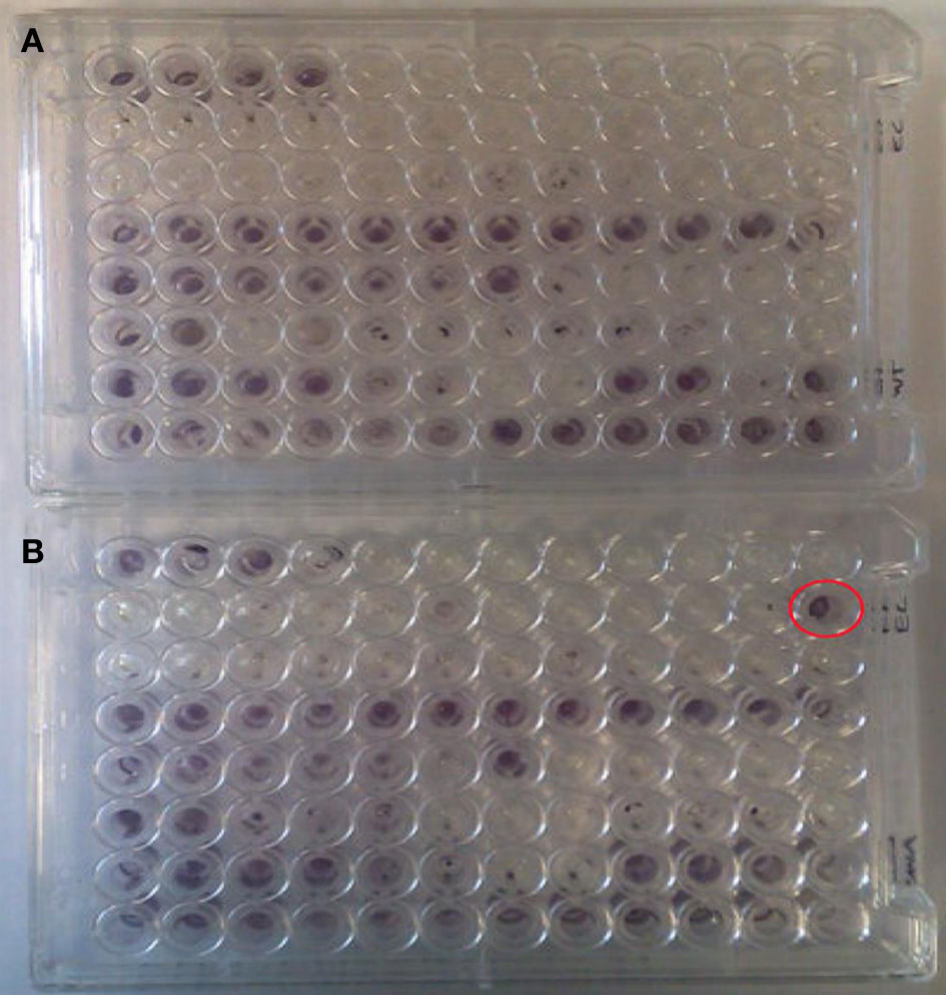
**Appearance of PM 9 plates after incubation for 24 hours at 37°C. (A)** Control EPI300::pCC1FOS and **(B)** SMG 9. PM plates measure cellular respiration colorimetrically via reduction of a tetrazolium dye with electrons from NADH generated during the process of respiration. Strongly metabolized substrates generate a more intense purple color. Development of a strong purple color can be seen in well B12 in Figure 2B (circled in red), which was inoculated with SMG 9, while no color development is visible in B12 of the control plate. This indicates SMG 9 has a greater ability to transport and utilise L-carnitine compared to the EPI300::pCC1FOS host strain.

**Figure 3:**
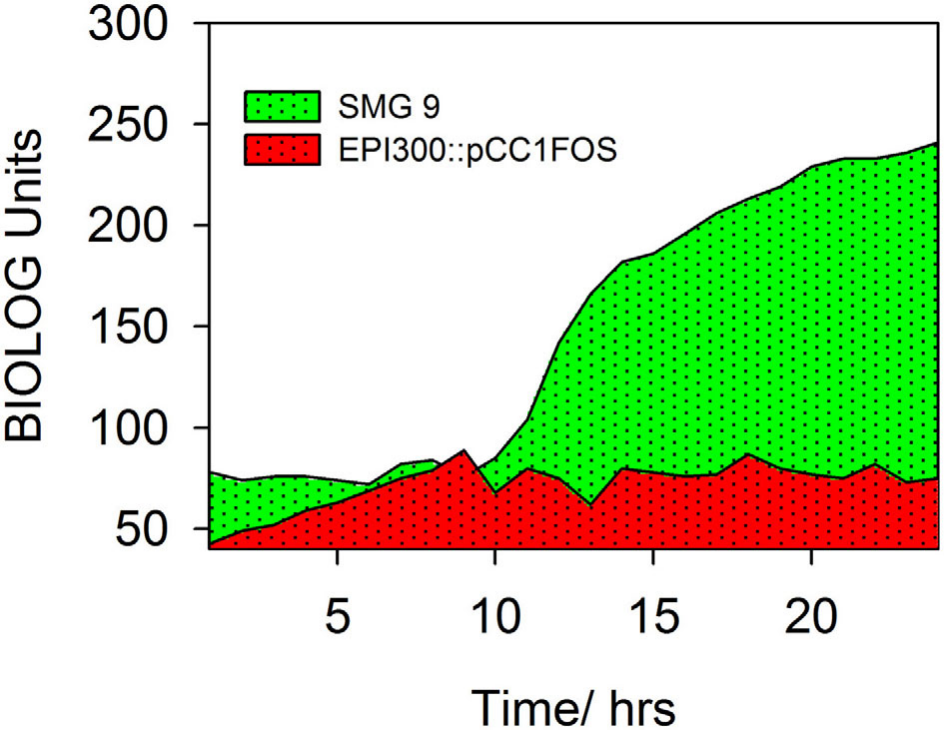
**Kinetic data measured by BIOLOG Omnilog system**. Color formation within each well was measured by BIOLOG's Omnilog machine, which produces a color coded graph. Kinetic data from two clones can be compared. EPI300::pCC1FOS is shown in red and that from SMG 9 is shown in green. The green color indicates more rapid metabolism by SMG 9 under the conditions in the well (6% NaCl + L-carnitine).

A number of clones initially identified from the metagenomic library were chosen at random and phenotypically screened using PM 9 osmolytes plate from BIOLOG. The layout and contents of PM9 can be viewed at http://www.biolog.com/pdf/pm_lit/PM1-PM10.pdf. One clone, SMG 9, gave a positive result under one of the 96 different conditions tested. It was found that SMG 9 had an increased metabolic response (causing a reduction of the tetrazolium dye to generate a purple color, Figure 2A) compared to the cloning host containing an empty fosmid vector, *E. coli* EPI300::pCC1FOS (Figure 2B) in 6% w/v NaCl supplemented with L-carnitine. The color formation within each well was measured by BIOLOG's Omnilog machine, which produces a color-coded graph. A comparison of the kinetic data output from SMG 9 and EPI300::pCC1FOS can be seen in Figure 3.

### Confirmatory experimentation of PM analysis

**Figure 4:**
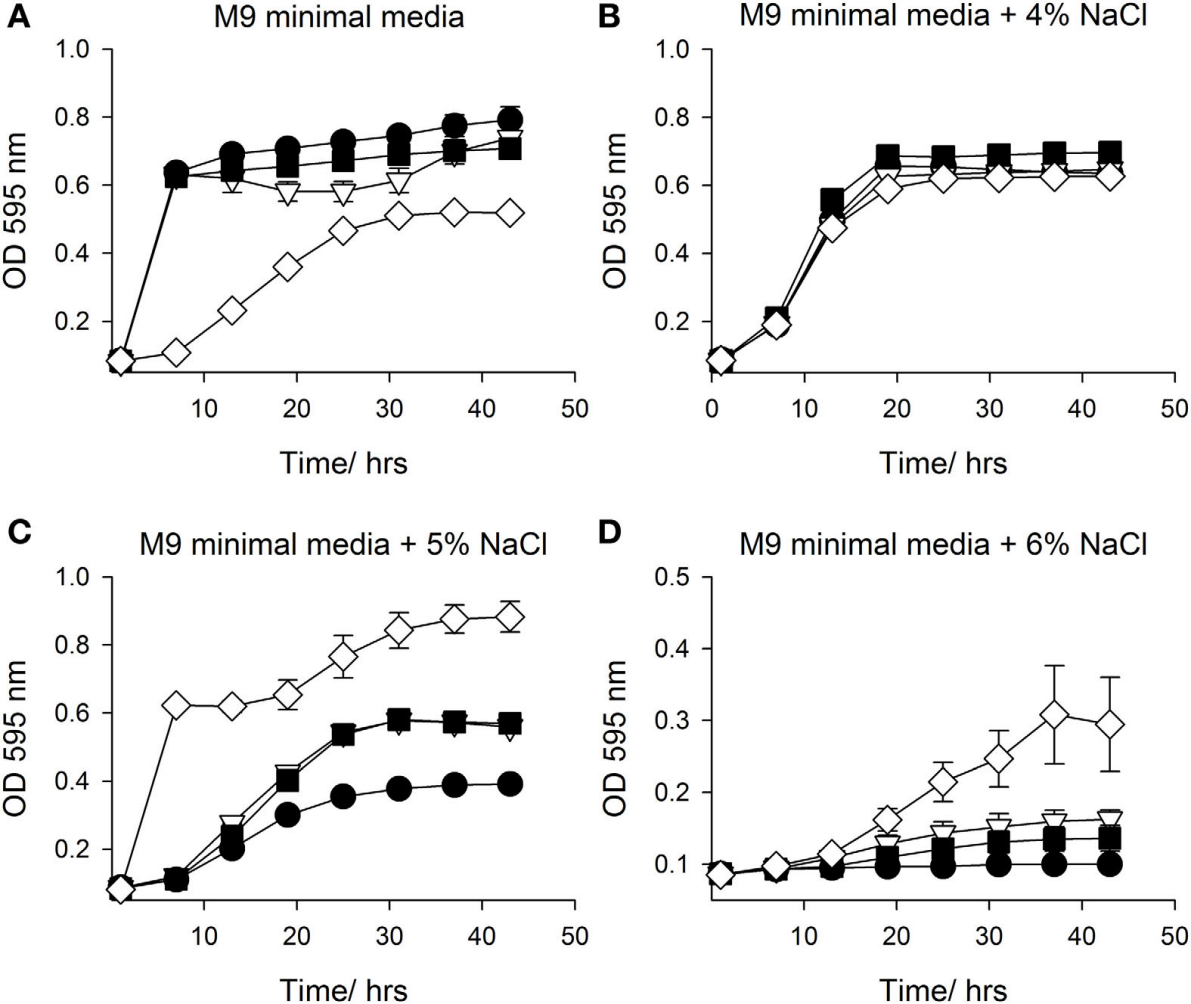
**Growth in M9 minimal media with NaCl +/− 1mM L-carnitine**. Growth of *E. coli* EPI300::pCC1FOS and SMG 9 in **(A)** M9 minimal media, **(B)** M9 minimal media + 4% NaCl, **(C)** M9 minimal media + 5% NaCl and **(D)** M9 minimal media + 6% NaCl. **Legend:** *E. coli* EPI300::pCC1FOS (• closed circle); SMG 9 (▽ open triangle); *E. coli* EPI300::pCC1FOS + 1mM L-carnitine (■ closed square); SMG 9 + 1 mM L-carnitine, (◊ open diamond).

In an attempt to confirm and replicate the result from the PM analysis, SMG 9 and EPI300::pCC1FOS were grown in M9MM containing various concentrations of NaCl (0–6% w/v) and supplemented with 1 mM L-carnitine when appropriate. Figure 4A shows growth of both clones in M9MM in the presence and absence of 1 mM L-carnitine. In the presence of L-carnitine, SMG 9 displays a growth defect, while growth is similar under all other conditions. The growth defect is alleviated when grown at 4% w/v NaCl + 1 mM L-carnitine and there is no difference in growth between clones either in the presence or absence of L-carnitine (Figure 4B). At 5% w/v NaCl however, SMG 9 has a significant growth advantage compared to EPI300::pCC1FOS both in the presence and absence of 1 mM L-carnitine (Figure 4C). The positive effect of L-carnitine on the growth of SMG 9 is evident with cells entering logarithmic phase growth sooner and reaching a much higher final optical density (OD_595 nm_). A similar, positive effect for L-carnitine on growth of SMG 9 is also seen at 6% w/v NaCl (Figure 4D).

### Sequencing of SMG 9 fosmid insert and analysis

Fosmid SMG 9 was fully sequenced (454-pyrosequencing) and assembled by GATC Biotech. This generated a total of 2.3 × 10^6^ base pairs of sequence data in 6939 sequencing reads. The average read length was 334 base pairs and coverage of 64.5× was achieved. Following vector trimming the length of the insert was approximately 36.5 kb and the %G+C content was 49.43%. Twenty-four putative open reading frames were predicted using Softberry's FGENESB, bacterial operon and gene prediction software (www.softberry.com) (Mavromatis et al., 2007). Translated nucleotide sequences were functionally annotated by homology searches using the BLASTP program to identify homologous sequences and determine their taxonomic origin. All but two of the genes encoded proteins with high identity (98–100% at the amino acid level) to *Bacteroides* sp. CAG:545. A list of the genes on SMG 9, their encoded functions and putative domains are presented in Table 2. The full fosmid insert sequence of SMG 9 has been submitted to GenBank and assigned the accession number, KJ524644.

**Table 2:** **Proteins predicted to be encoded on SMG 9 fosmid insert**.

**Gene #**	**Putative encoded function**	**# a.a**	**Closest hit organism**	**% Coverage**	***e*-value**	**% ID**	**Domains**
1	ATP-dependent chaperone ClpB	554	*Bacteroides* sp. CAG:545	97	0.00E + 00	99	COG0714; AAA ATPase
2	Preprotein translocase SecG subunit	121	*Bacteroides* sp. CAG:545	100	3.00E-77	100	SecG
3	Putative uncharacterized protein	187	*Bacteroides* sp. CAG:545	100	3.00E-134	100	None detected
4	Putative uncharacterized protein	177	*Bacteroides* sp. CAG:545	86	3.00E-108	99	LptE
5	Transcriptional regulator	432	*Bacteroides* sp. CAG:545	100	0.00E + 00	99	AAA ATPase; σ ^54^ interaction domain; HTH_8 bacterial reg protein, Fis family domain
6	Putative uncharacterized protein	545	*Bacteroides* sp. CAG:545	100	0.00E + 00	99	TadD
7	Putative uncharacterized protein	1015	*Bacteroides* sp. CAG:545	100	0.00E + 00	99	SecD, SecF
8	OmpA/MotB domain protein	618	*Bacteroides* sp. CAG:545	71	0.00E + 00	99	PD40, similar to WD40 domain
9	Putative uncharacterized protein	155	*Bacteroides* sp. CAG:545	100	4.00E-106	100	NfeD
10	uPF0365 protein AL1_06760	317	*Bacteroides* sp. CAG:545	100	0.00E + 00	99	YdfA_immunity superfamily
11	Putative uncharacterized protein	142	*Bacteroides* sp. CAG:545	100	1.00E-97	98	Lipocalin_4
12	Subtilisin-like serine protease	678	*Bacteroides* sp. CAG:545	100	0.00E + 00	99	Peptidase_S8_S53 superfamily
13	Uncharacterized protein	812	*Bacteroides* sp. CAG:545	99	0.00E + 00	99	None detected
14	RagB/SusD family protein	547	*Bacteroides* sp. CAG:545	100	0.00E + 00	99	Two SusD superfamily
15	Outer membrane receptor for ferrienterochelin and colicins	1068	*Bacteroides* sp. CAG:545	100	0.00E + 00	100	Can_B2; Plug; OM channel; OMP_RagA_SusC
16	Alpha-L-fucosidase-like	513	*Bacteroides* sp. CAG:545	100	0.00E + 00	98	COG3669; Alpha_L_fucos; F5_F8_Type_C
17	Putative uncharacterized protein	446	*Bacteroides* sp. CAG:545	100	0.00E + 00	99	DHQ_FE-ADH (Dehydroquinate iron aldehyde dehydrogenase)
18	Major facilitator superfamily MFS_1	487	*Bacteroides* sp. CAG:545	100	0.00E + 00	100	MFS; UhpC, sugar phosphate permease
19	ThiF family protein	242	*Bacteroides* sp. CAG:545	100	4.00E-174	98	YgdL_like
20	Putative uncharacterized protein	649	*Bacteroides* sp. CAG:545	100	0.00E + 00	98	Glyco_hydro_97
21	DNA mismatch repair protein MutS	894	*Bacteroides* sp. CAG:545	98	0.00E + 00	99	PRK05399; MutS-I; MutS_II; MutS_III; ABC_MutS_1
22	Glycosyl transferase group 1	378	*Bacteroides* sp. CAG:545	100	0.00E + 00	99	RfaG
23	No significant similarity found	68	N/A	N/A	N/A	N/A	N/A
24	Hypothetical protein Fjoh_3657	162	*Flavobacterium johnsoniae* UW101	80	4.00E-25	40	AdkA, archaeal adenylate kinase

*Functional assignment is based on BLASTP of amino acid sequences predicted by Softberry's FGENESB. Abbreviations: # a.a, number of predicted amino acids; %ID, % identity at the amino acid level; N/A, not applicable*.

### Cloning of *mfsT* and *sdtR* genes

Following initial inspection of the encoded proteins on SMG 9, the presence of an L-carnitine or general osmoprotectant transporter, nor indeed any protein with a functional link to carnitine metabolism was not immediately obvious. Transposon mutagenesis was attempted in order to create a knock-out mutant; this however, proved unsuccessful. Two genes (gene 5 and gene 18), which we felt may be likely to have a possible role in L-carnitine utilization based on bioinformatic analysis were cloned in isolation to further examine the phenotype. Genes 5 and 18 were annotated *sdtR* for sigma-dependent transcriptional regulator and *mfsT* for major facilitator superfamily transporter, respectively.

**Figure 5:**
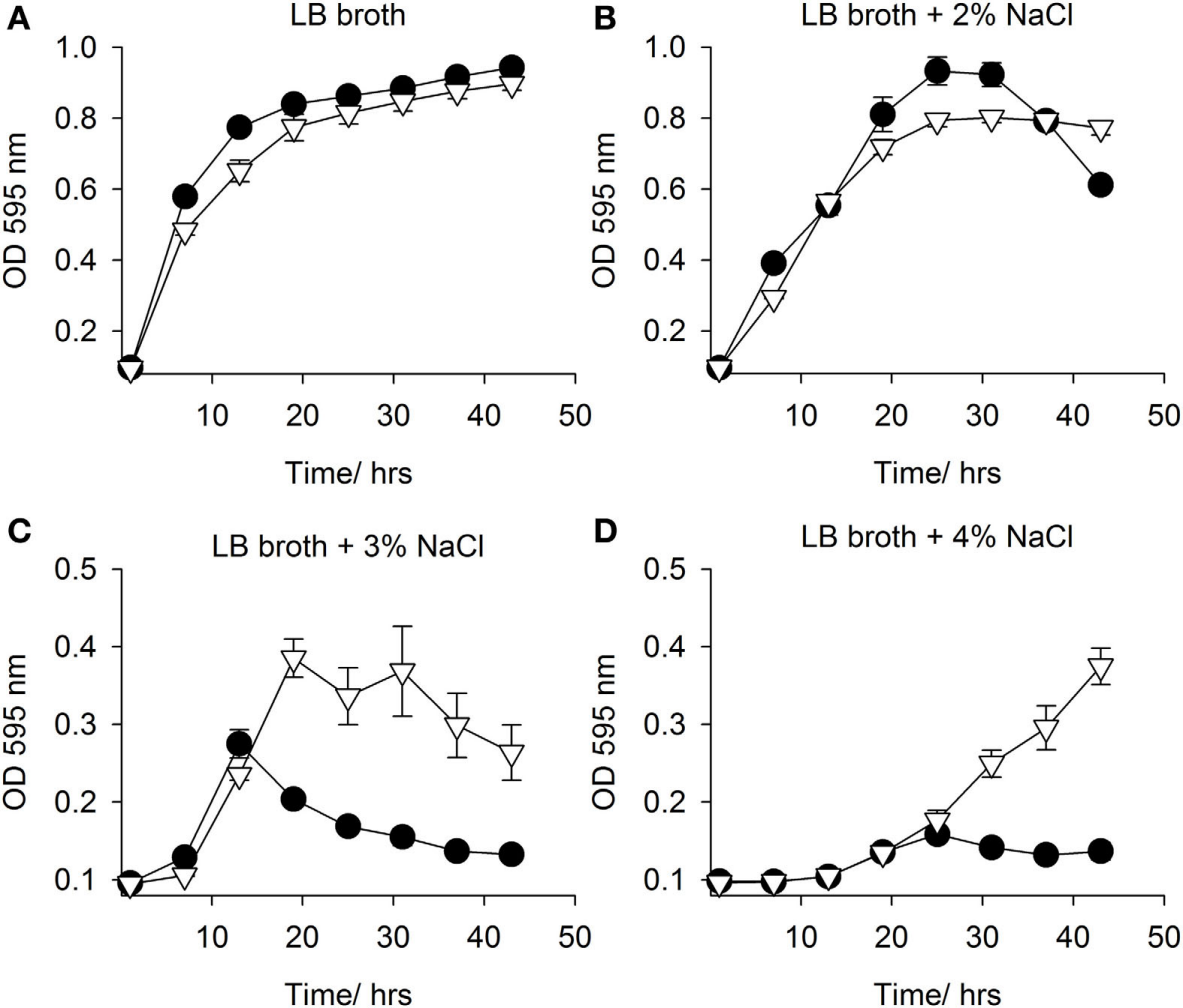
**Growth in LB broth with NaCl**. Growth in of *E. coli* MKH13::pCI372 (• black circle) and MKH13::pCI372-*sdtR* (▽ open triangle) in **(A)** LB broth and LB broth supplemented with **(B)** 2% NaCl, **(C)** 3% NaCl and **(D)** 4% NaCl.

**Figure 6:**
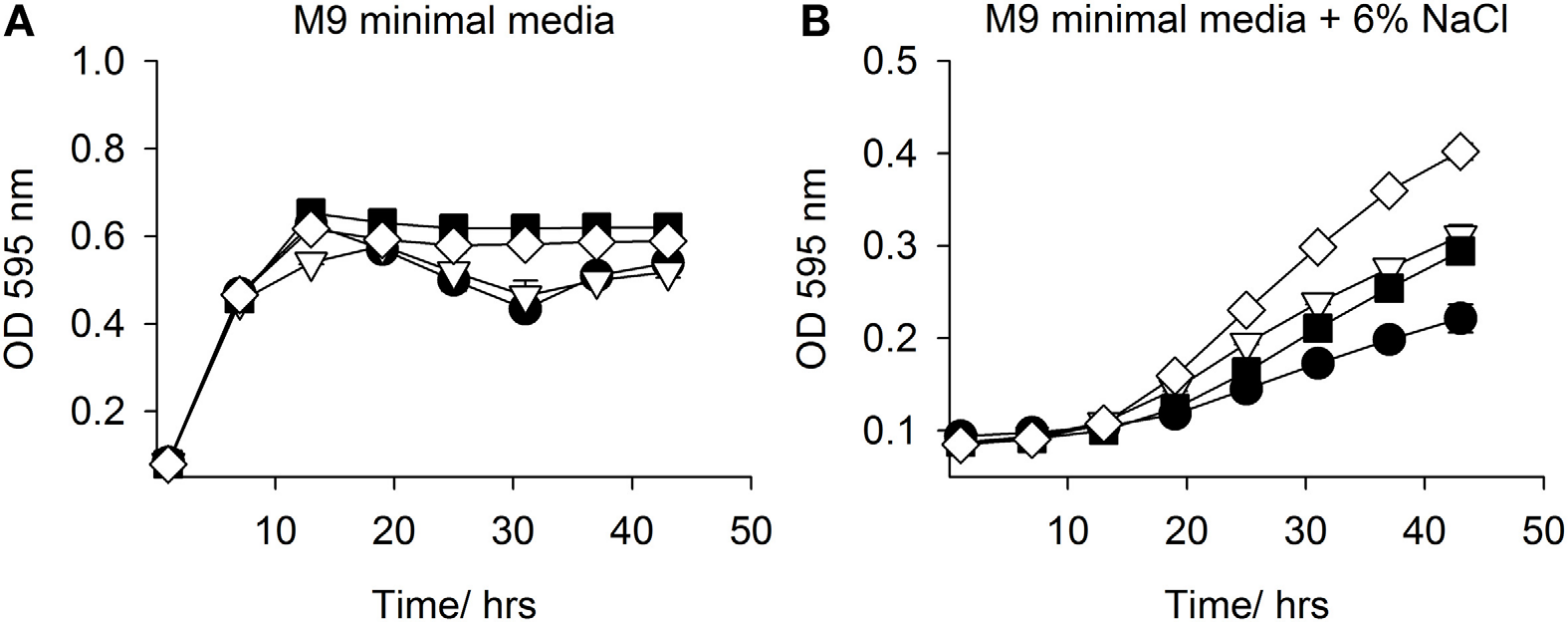
**Growth in M9 minimal media with NaCl +/− L-carnitine**. Growth of EPI300::pCI372 and EPI300::pCI372-*sdtR* in **(A)** M9 minimal media and **(B)** M9 minimal media + 6% NaCl. **Legend:** *E. coli* EPI300::pCI372 (• black circle); EPI300::pCI372 + 1 mM L-carnitine (▽ open triangle); EPI300::pCI372-*sdtR* (■ closed square); EPI300::pCI372-*sdtR* + 1mM L-carnitine, (◊ open diamond).

Both *mfsT* and *sdtR* were cloned in the vector pCI372 and expressed in both *E. coli* EPI300 and the osmosensitive strain *E. coli* MKH13 (Haardt et al., 1995). The effect of each gene on the growth of each strain under salt stress in the presence and absence of L-carnitine was assessed. The *mfsT* gene had no effect on growth under any of the conditions tested (data not shown). The *sdtR* gene on the other hand conferred a significant salt tolerance phenotype to *E. coli* MKH13 when grown in media supplemented with both 3 and 4% w/v NaCl (Figures 5C,D, respectively), while growth was similar in media lacking NaCl and in media supplemented with 2% w/v NaCl (Figures 5A,B, respectively). Cloning and expression of *sdtR* in EPI300 resulted in an increase in salt tolerance compare to wild-type EPI300 carrying an empty copy of the plasmid pCI372. Addition of 1 mM L-carnitine increased the growth rate and final optical density of both strains, but its effect on the *sdtR*^+^ strain was not significant relative to the EPI300::pCI372 control (Figures 6A,B).

Although *sdtR* did confer a salt tolerance phenotype, neither of the cloned genes replicated the original phenotype related to L-carnitine. We re-examined clone SMG 9 and carried out further studies, however these revealed that SMG 9 had lost the carnitine-associated phenotype seen originally and unfortunately, it was therefore not possible to identify the gene(s) responsible.

## Discussion

In the present study we have identified a novel salt tolerance gene from a metagenomic library clone from the human gut microbiome using a combined functional metagenomic and PM approach. The clone, SMG 9, was identified from a previous library screen to identify salt tolerant clones (Culligan et al., 2012) and was further characterized in this study using PM osmolyte plates. From the PM screen, SMG 9 showed an increased metabolic profile in the presence of 6% NaCl + 1 mM L-carnitine compared to the control strain carrying an empty fosmid vector (EPI300::pCC1FOS), indicating this clone could utilize or transport L-carnitine. Experiments to confirm the findings of the PM assay showed that SMG 9 displayed an increased growth profile at 5% and 6% NaCl in the presence of 1 mM L-carnitine compared to controls, similar to observations in the PM assay. Transposon mutagenesis was attempted to create phenotypic knock out mutants, using the EZTn*5* system (Epicentre Biotechnologies; Goryshin and Reznikoff, 1998) but this proved unsuccessful. This may be due to the presence of a gene encoding a DNA repair protein MutS on the fosmid insert, which has been associated with transposon excision (specifically Tn5 and Tn10) (Lundblad and Kleckner, 1985).

Next generation sequencing of the full fosmid insert of SMG 9 and functional assignment of the encoded proteins using BLASTP revealed sequences shared highest genetic identity to *Bacteroides* sp. CAG:545. Species of *Bacteroides* are commonly found in the human gut, where the resident microbiota is largely composed of species from two dominant phyla, the *Bacteroidetes* and *Firmicutes* (Qin et al., 2010). The %G+C content of the SMG 9 insert was 49.44% which close to the reported range of 40–48% for genomes of species of *Bacteroides* (Shah, 1992). Sequencing and subsequent functional annotation did not reveal any obvious genes related to known L-carnitine transport or utilization systems, suggesting a novel mechanism may be involved. We conducted further bioinformatic analysis of the encoded proteins in an attempt to identify any link to salt tolerance or osmoprotectant transport. Gene 5 (*sdtR*) is predicted to encode a σ ^54^-dependent transcriptional regulator, which contains a number of domains (see Table 2), including a helix-turn-helix 8 (HTH_8), Fis-family protein domain, while gene 18 (*mfsT*) is predicted to encode a major facilitator superfamily (MFS) protein with an UhpC sugar phosphate permease domain. Both genes were chosen for further study and cloning as Fis is a regulatory protein involved the regulation of proline (another important osmoprotectant) uptake (Xu and Johnson, 1995, 1997; Typas et al., 2007), while we further reasoned that *sdtR* could be regulating host EPI300 genes, contributing the L-carnitine-associated phenotype, while MFS transporters also play a role in osmoprotectant uptake (Culham et al., 1993; Haardt et al., 1995; Pao et al., 1998). Despite the presence of a sugar phosphate permease domain, indicating sugar transport, MFS transporters are known to have a diverse substrate range (Pao et al., 1998; Saier, 2000; Law et al., 2008).

The *mfsT* gene did not confer a salt tolerance or the L-carnitine associated phenotype to transformed cells (data not shown). The *sdtR* gene also did not confer the L-carnitine associated phenotype to *E. coli*, but did however confer an increased salt tolerance phenotype. *sdtR* therefore represents a novel salt tolerance gene and most likely functions by influencing expression (either positively or negatively) of host *E. coli* genes, although further work, comprising expression studies and microarray analysis, will be required to elucidate the genes involved as transcriptional regulators can influence a wide variety of genes. Transcriptional regulators are commonly involved in the response different stresses in bacteria (Hengge-Aronis et al., 1991; Cheville et al., 1996; Battesti et al., 2011; Hoffmann et al., 2013), while σ ^54^ (RpoN) has been shown to play a role in osmotolerance in *Listeria monocytogenes* (Okada et al., 2006).

The loss of the carnitine-associated phenotype of SMG 9 prevented further characterization of this clone and ultimately the identification of the gene(s) responsible. It is difficult to pinpoint the cause of this phenotypic reversion, but a clue may be evident from Figure 4A, where a growth defect for SMG 9 is apparent when grown in M9MM + 1 mM L-carnitine. This indicates L-carnitine may be increasing the metabolic load on the cell and this metabolic stress is only relieved in the presence of NaCl, when L-carnitine may be utilized efficiently in an osmoprotective capacity. If the gene is constitutively expressed, a mutation may have occurred to counteract this phenomenon. The presence of a gene encoding MutS may also be relevant as mutations to MutS can result in a mutator phenotype in *E. coli* cells (Wu and Marinus, 1994). Furthermore, it is possible the original SMG 9 clone acquired a mutation on the fosmid insert that conferred the carnitine-associated phenotype and a subsequent suppressor mutation occurred to silence this mutation, returning the clone to its original phenotype.

In conclusion, we have identified a novel salt tolerance gene from the human gut microbiome using a combined functional metagenomic and PM approach. The gene originates from a species of *Bacteroides* and encodes a putative transcriptional regulator. Overall this study demonstrates the utility of functional metagenomics and phenomics for novel gene discovery and functional characterization of metagenome-derived clones.

### Conflict of interest statement

The authors declare that the research was conducted in the absence of any commercial or financial relationships that could be construed as a potential conflict of interest.
